# Entraining neurons via noninvasive electric stimulation improves cognition

**DOI:** 10.1371/journal.pbio.3000931

**Published:** 2020-10-22

**Authors:** Mircea van der Plas, Simon Hanslmayr

**Affiliations:** 1 School of Psychology, University of Birmingham, Birmingham, United Kingdom; 2 Centre for Human Brain Health, University of Birmingham, United Kingdom; 3 Institute for Neuroscience and Psychology, University of Glasgow, Glasgow, United Kingdom

## Abstract

Transcranial Alternating Current Stimulation (tACS) is a method that injects rhythmic currents into the human brain via electrodes attached to the scalp of a participant. This technique allows researchers to control naturally occurring brain rhythms and study their causal relevance for cognition. Recent findings, however, cast doubts on the effectiveness of tACS to stimulate the brain and its mode of action. Two new studies by Vieira and colleagues and Marchesotti and colleagues reported in the current issue report promising new results in showing that tACS can entrain single neuron activity and improve reading abilities in dyslexic individuals.

## Abstract 2

This Primer explores the implications of two recent studies, one showing that transcranial alternating current stimulation (tACS) entrains single neurons in the brain, and the other that 30 Hz stimulation (via tACS) improves reading abilities in individuals with dyslexia. These studies show how tACS can affect neural processing in the brain in order to improve cognition in humans.

Cognitive neuroscientists nowadays have an arsenal of methods at their disposal to study the human brain. Methods such as scalp or intracranial electroencephalography (EEG/iEEG), magnetoencephalography (MEG), and functional magnetic resonance imaging (fMRI) record brain activity as it labors tirelessly in order to make sense of the world, enabling researchers to establish correlations between patterns of brain activity and behavior. While these studies have without doubt generated a wealth of knowledge, this approach limits neuroscientists to establishing correlational links, i.e., brain state X co-occurs with behavior Y. Notably, this is different from and considerably weaker than establishing a causal link, i.e., brain state X causes behavior Y. Establishing such causal relationships between brain patterns and behavior requires a method that directly changes the brain’s activity and affects behavior accordingly.

One set of brain patterns that have often been assumed to have functional relevance for a wide variety of processes are neural oscillations [1,2]. Brain oscillations are usually categorized according to their frequency into one of many different bands, each associated with a multitude of processes within the brain. In order to draw any causal links between neural oscillations and behavioral processes, one has to somehow directly affect these neural oscillations. If one can both improve and deteriorate performance for a given task by manipulating these neural oscillations, one can make strong claims about its relevance for behavior.

Transcranial Alternating Current Stimulation (tACS) is an electrical stimulation method that is often used with the aim of manipulating these brain oscillations directly [3–6]. This relatively simple procedure involves attaching at least two electrodes on the scalp, through which an alternating current (AC) is administered. Depending on the placement of the electrodes, the currents’ electrical field can affect the underlying neurons and bias their firing with subthreshold polarity changes in the surrounding cerebrospinal fluid (CSF) in an oscillatory fashion (see Fig 1A). For example, if the surroundings of a neuron become more positively charged compared to its insides, the neuron will require a larger change in polarity to reach the threshold for an action potential, while a less positive charge will lower the threshold for an action potential. Since the direction of current flow is switched at a fixed rate, tACS biases the firing of the targeted neural population such that action potentials co-occur at certain time windows. In the human brain, co-occurrence of firing is evident in oscillatory activity, which is naturally present at many different rhythms [1]. TACS targets these natural brain oscillations in order to shift their phase to the phase of the applied stimulation, which is referred to as “entrainment” [7]. It is important to highlight that the stimulation resulting from tACS is merely assumed to affect the timing/synchronicity of neural firing but not directly affect the ongoing firing rate (see Fig 1B and 1C for an illustration). The window of how much one can affect a natural brain oscillation depends on both the field strength and frequency of the applied current. The lower the strength of the applied electric field, the smaller the window of frequencies one may entrain. This concept can be visualized with a diagram called the Arnold Tongue (see Fig 1D) [8,9].

**Fig 1:**
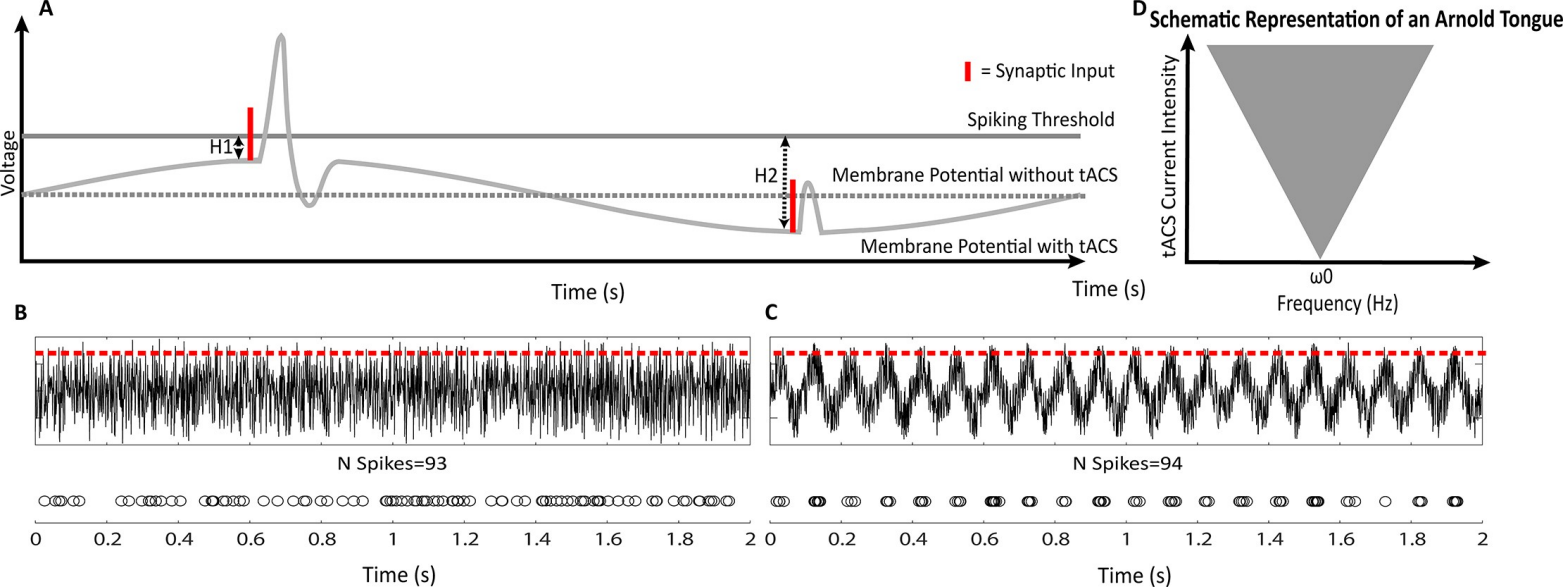
**(A)** Schematic Illustration of the commonly assumed mechanism of tACS. A typical sinusoidal cycle of tACS stimulation is illustrated, onto which action potentials of a hypothetical neuron are superimposed. At the peak of the wave, the current is assumed to raise the general excitability of neurons, pushing a given neuron closer to its firing threshold (solid black line). At the trough of the wave, the current pulls the neuron away from its firing threshold; therefore, the same synaptic input (visualized as a red line) might excite a given neuron sufficiently for an action potential in one state but not the other. **(B–C)** Simplified representation of the effects of sinusoidal stimulation on spike timings and spike rate as a result of an illustrative simulation. **(B)** Simulation of random noise, as a representation of ongoing activity. The red dotted line represents the threshold that has to be passed for an “action-potential.” Scatterplot below shows the amount of spikes and their timings. **(C)** Same as in B but with a sinusoidal on top of the random noise wave. While B and C have a very similar absolute amount of spikes, the distribution of spike-timings is random in B but is patterned according to the peaks of the sinusoid in C, just as one would expect from actual tACS. **(D)** Simplified schematic of an Arnold Tongue. The grey area indicates the frequency range that can be successfully entrained between two oscillators (in this case the tACS stimulation and the neural activity). With increasing amplitude (*ε*), a wider range of frequencies (*ω*) around the naturally occurring frequency (*ω*0) in the brain can be entrained. tACS, transcranial Alternating Current Stimulation.

Due to its potential in directly altering ongoing oscillations in the brain, tACS quickly became an attractive method in a variety of contexts. However, much of the early research predated a deeper understanding of the method, resulting in tACS frequently being used suboptimally, leading to many null effects and failures to replicate positive results. This has frustrated the field and led many to question whether tACS can actually directly entrain neural activity [10–12]. One of the major criticisms resulted from an influential study applying tACS on human cadavers, which found that the electric currents commonly induced by Transcranial Electrical Stimulation (TES) were too weak to actually affect neural firing [13]. Further research on in vivo primate models and humans suggests that stimulation in the range of 1 mA peak-to-baseline (i.e., 2 mA peak-to-peak) can induce electrical fields (>0.2 mV/mm) large enough to affect spike timing, in accordance with existing models of electrical conductivity in the brain [14,15]. This does cast doubt on many early studies using much lower stimulation intensities. It should, however, be noted that other factors, like electrode montage or distance to stimulation site, and not just intensity also play an important role. The above-cited studies are consistent with findings showing that tACS, applied in the range of 1 mA peak-to-baseline (i.e., 2 mA peak-to-peak), can affect spike timing of single neurons and neural adaptation in primates [16,17]. However, one problem with such intensities is that they also induce sensory effects (which is why earlier studies used lower intensities in the first place). This raises the additional concern that the mode of action of tACS is not through the current affecting neuronal populations in the brain directly but rather through its side effects, such as the somatosensory stimulation or stimulation of the retina, or indirect stimulation through cranial nerves [18–20]. A recent study by Asamoah and colleagues found that transcutaneous stimulation of peripheral nerves in the skin could entrain neurons in a rat model [21]. This finding was further supported by an additional experiment in the study in which anesthetic cream removed any tremor effects related to tACS in human participants, further linking somatosensory sensation and behavioral effects.

The study published in this issue by Vieira and colleagues can be seen as a direct response to the previously discussed research on the mechanisms of tACS [22]. The study further explores the mechanisms of tACS, by measuring neural firing in a primate model while systematically varying the parameter space, and its dependency on sensory stimulation. Vieira and colleagues report the effects of tACS on single neuron activity independent of somatosensory perception, supporting the idea that tACS can directly affect neural populations in certain conditions, not merely through indirect sensory means. One should note, however, that they were not able to completely disentangle direct effects of tACS from the possibility of affecting the populations indirectly through cranial nerves. However, not much research has focused on the relevance of cranial nerves on tACS, and this issue requires more attention by future research in general. Nevertheless, this study adds some compelling evidence, making the case for the capability of tACS to directly entrain neuronal populations in certain conditions.

Neural oscillations have been linked to a wide variety of phenomena, not only in the healthy brain but also in pathological cases [23,24]. Thus, research into the mechanisms of tACS helps researchers to fine-tune their parameters so that tACS may be used in a very targeted fashion to alter brain function, not just for research purposes but for clinical purposes as well. For example, neural oscillations have previously been implicated in language processing [25]. Thus, it would make sense that individuals with language processing issues, such as individuals suffering from dyslexia, might benefit from some kind of tACS stimulation. The study published in this issue by Marchesotti and colleagues provides an example for the potential of tACS to improve brain functioning in impaired individuals, as well as understanding ongoing processes in the brain [26]. Marchesotti and colleagues were able to improve reading performance in individuals suffering from dyslexia by applying targeted tACS at 30 Hz over the left auditory cortex, thus causally implicating the importance of oscillatory activity at 30 Hz for phonological processing. This important finding opens a potential avenue to aid individuals struggling with dyslexia and is a good example of how targeted tACS can be used for clinical treatments in addition to the research purposes.

Even though the mechanisms of tACS are still not completely understood, many studies are being performed in order for tACS to be used to the most of its potential. The study by Vieira and colleagues on a primate model has further explored the mechanism of tACS and its mode of action. Results from these types of studies into the mechanisms of tACS critically help tACS researchers in choosing the right parameters for their studies. In addition, such studies allow tACS to be used more effectively, by fine-tuning tACS to specific hypotheses, as demonstrated by Marchesotti and colleagues in work published in this issue [26]. Research efforts such as these will ultimately answer causal questions of brain oscillations and provide a means for novel treatments for a variety of disorders.

## Abbreviations

AC: alternating current
CSF: cerebrospinal fluid
EEG: electroencephalography
fMRI: functional magnetic resonance imaging
iEEG: intracranial electroencephalography
MEG: magnetoencephalography
tACS: transcranial Alternating Current Stimulation
TES: Transcranial Electrical Stimulation
